# Interesting Analyses of Articles in Foreign Journals

**Published:** 1864-12

**Authors:** 


### INTERESTING ANALYSES OF ARTICLES IN FOREIGN JOURNALS.

*Observations on the Operation for the Division of the Ciliary Muscle.* By Henry Hancock, F. R. C. S.

During the three years which have elapsed since the operation for the division of the ciliary muscle was first introduced to the notice of the profession, it has been employed in various affections of the eye. The diseases in which it has proved most successful have been kerotitis, sloughing of the cornea, staphyloma, dense opacity of the cornea, (in some cases of several years' duration,) and conical cornea; also in certain forms of amaurosis; in acute and chronic glaucoma, and in posterior staphyloma and myopia. Before, however, I enter upon the detail of these cases, I would explain the grounds upon which I have recommended this operation, inasmuch as some gentlemen who have written upon the subject have very erroneous opinions on the matter, and attribute ideas to me which I by no means entertain.

Although considerable attention has been paid to the structure and use of the ciliary muscle, no one has hitherto sufficiently insisted on the important influence which it exerts, in disease, upon the circulation and consequent nutrition of the eye-ball. The experiments of Cramer, Helmholtz, and H. Muller, whilst proving the value of this part in the "accommodation of the eye," show also that, in health, it possesses both elasticity and contractility; and whilst the experiments of Helmholtz demonstrate the influence which it exerts in the adaptation of the lens to the accommodation of the eye, we cannot overlook the influence which it must also exert over the cornea, sclerotica, choroid, and iris, in adapting them likewise, especially when its connection with these coats of the eye is borne in mind. Heinrich Muller distinguishes two sets of muscular fibres in the ciliary muscle: *external* or *radial*, springing from the inner wall of Schlem's canal, and passing outwards and backwards, to be inserted both in the schlerotic and choroid; an *internal* or *circular*, running parallel with the corneal margin, and situated principally in the antero interior part of the muscle, near the insertion of the iris. As a whole, we find it closely connected with the line of junction between the cornea and schlerotica, the choroid and iris; and receiving the middle portion of the posterior elastic layer of the cornea, whilst the most anterior portion forms distinct columns, constituting the pillars of the iris, (Bowman,) or "ligamentum iridis pectinatum (Huck). "It is continued with the iris, and is closely attached to the circular sinus of Schlem, connecting the iris at this point and constituting a bond of union between it and the ciliary processes. The retina likewise terminates in its ora senata at the posterior edge of this muscle. The long and several of the anterior ciliary arteries pass through it, as well as many of the posterior ciliary arteries, in their passage from the choroid to the iris; whilst the choroidal veins, having reached the ciliary muscle, turn with a sharp curve along it, and, uniting, form a nearly straight horizontal vessel, of considerable size, along its posterior edge. The ciliary nerves also run through it, ramifying freely throughout its substances." (Nunneley: "O gans of Vision," p. 168). And although absorbents have not been demonstrated in this structure, it may fairly be inferred that this arises from their extreme minuteness, rather than from their non-existence.

From the above description we gather that the so-called ciliary muscle is a body possessing, in the natural state, both elasticity and the power of contractility, exerting the power of accommodation of the eye to various foci, and permitting the transmission of blood-vessels, nerves, and, in all probability, of absorbents, through it, without interruption to their functions.

In a paper on Glaucoma (The Lancet, January 26th, 1861), Mr. Nunneley writes—"Mr. Hancock believes the disease does not depend upon hypersecretion of the vitreous humor owing to disease of the choroid coat, but that it essentially depends upon continuous spasm of the ciliary muscle, which, according to him, induces the hardness of the globe of the eye." Whilst Mr. Haynes Walton, in the last edition of his work on 'Surgical Diseases of the Eye," observes—"It is not probable, I think, that such a muscle as the ciliary could contract with sufficient force to groove the hard and stony eye-ball of glaucoma, still less likely is it that such spasm could be continuous."

I have never believed, nor have I ever stated, that glaucoma is caused by spasm of the ciliary muscle, whether persistent or temporary. I have stated that, in my opinion, the opthalmoscopic and pathological appearances of the *blood-vessels* were greatly enhanced by, if not in some instances entirely due to, the obstruction of the circulation caused by the undue and excessive constriction exerted on them by the spasmodic or *extreme contraction* of the ciliary muscle; but I have never, as yet, said anything about the ciliary muscle inducing hardness of the globe of the eye, nor of its contracting with sufficient force to groove the "hard and stony eye ball of glaucoma."

I believe that glaucoma is not caused by hypersecretion of the vitreous humor; but that it has its origin in some peculiar condition of blood, considered by most ophthalmic surgeons as gouty or rheumatic—a view of the question most ably supported by M. Canton, in his admirable papers "On Atrophy and Degeneration of the Arteries," and confirmed by the writings of Lawrence, Siehel, Mackenzie, Tyrrel, Bell, as well as by the dissections of Eble and Rosas; that the muscular fibres and blood-vessels become implicated, as sooner or later do the heart and blood-vessels, in that disease; that the ciliary muscle, losing its elasticity and contractility, is converted into a rigid, unyielding cord. The eye is consequently deprived of its accommodating power, and the blood-vessels, nerves, and doubtless the absorbents, from their peculiar arrangement with reference to this muscle, being compressed, the circulation through these vessels is impeded; their coats, already weakened by the existing disease, yield, form aneurismal pouches, give way or become varicose. The parts supplied by these vessels, nerves, &c., are deprived of their nourishment, whilst the intraocular effusion or hypersecretion takes place subsequently to, and results from, these morbid changes.

In the case which I related in the Lancet for 1859, the attack came on so suddenly after exposure to strong light, without any premonitory symptoms, and was attended with so much acute pain, that I have no doubt the constriction, in this case at all events, resulted from spasmodic contraction perpetuated by the intensity and persistence of the disease. In other instances, however, when the disease progresses more slowly and insidiously, when there is gradual and progressive degeneration of structure, I can readily understand that the ciliary muscle should become implicated and converted into a rigid, unyielding, inelastic ring, from that cause, rather than spasmodic contraction; but although I firmly believe that these two conditions do exist, I am bound to admit that I cannot adduce any dissertations in support of this opinion, as in no instance has the operation for the division of the ciliary muscle been followed by any symptoms which would render extirpation of the eye-ball justifiable.

Mr. Haynes Walton seems to think that the hardness of the eye-ball precedes the constriction of the muscle, and that I believe the muscle, by its contraction, actually draws in the distended coats forming the sulcus met with in glaucoma and other diseases of the eye. On the contrary, I believe the contraction, whether spasmodic or otherwise, precedes the hardness, and that whatever influence the muscle exerts in these cases upon the coats of the eye is of a passive rather than of an active nature; that, in other words, it does not "groove" and draw the coats of the eye in, but that it prevents the coats of the eye expanding at this point; hence a given quantity of fluid effused within the eye-ball would render it more tense than when such a condition of the ciliary muscle does not obtain, and this may easily be verified by the following experiment: I took a piece of the intestine of an ox, and having placed an elastic India rubber ring around it, I forced in as much water as it would hold by means of a bladder syringe; of course, as the water was forced in the ring expanded, and the intestine became uniformly tense, and by measurement I found the quantity thrown in was forty two ounces. I then removed the India-rubber ring, and substituted a ring of string of the same size as the India rubber ring when not expanded, and I again injected as much water as I could; but as the string was inelastic, the gut was constricted at the point encircled by it, whilst the quantity of fluid measured only thirty-three ounces, so that the loss of elasticity diminished its capacity by nine ounces.

Whilst the division of the ciliary muscle, by doing away with constriction, diminishes the tension of the eye-ball and the consequent intra ocular pressure, it appears to me that the most important result of the operation is its effect upon the blood-vessels, nerves and absorbents, in removing the obstruction to the circulation and increasing nutrition; whilst a result of almost equal importance is the recovery of the accommodating power of the eye. It is, however, objected that these cannot, by any possibility, be effected by the operation which I recommend.

Mr. Nunneley observes (The Lancet, Jan. 26th, 1861):—"It cannot, however, be at all difficult to show that neither the theory nor the practice, as applicable to the theory of Mr. Hancock, can possibly be true. * * * Supposing the ciliary muscle were really hypertrophied and in a state of tonic spasm, how could it induce hardness of the whole globe of the eye, flattening of the cornea, and much intra-ocular pressure? * * * The fibres are not circular, but straight, and not more than one-seventh of an inch long. They can have no action whatever upon the schlerotic coat, to which they are not attached posteriorly. They cannot, therefore, render it hard." And he adds: "But even were this theory of compression by the ciliary muscle as true as it appears untenable, how could a simple puncture in the direction of its fibres interfere with the entire circle of its fibres? A broad, transverse incision in the direction of the corneal curve, by dividing the fibres, might act powerfully in proportion to the number of fibres divided; but that a simple momentary separation from each other of two or more adjoining parallel fibres, without any division of their structure (for such must be the effect of a fine, sharp, thin knife), could permanently arrest strong spasmodic action in an entire muscle spread over a large circle, it is impossible to conceive."

It is somewhat surprising that a gentleman professing to be an authority on the minute anatomy of the eye-ball should so decidedly commit himself to the assertion, that the fibres of the ciliary mussle are not "circular," but "straight." H. Muller has shown that the ciliary muscle consists partly of straight and partly of circular fibres, and it is difficult to understand what Mr. Nunneley actually means when he talks of the ciliary muscle as a set of unconnected straight fibres, not more than the seventh of an inch long, and then immediately describes it as "an entire muscle spread over a large circle." It will readily be granted that both these conditions cannot obtain. If it is merely an arrangement of unconnected, straight, radiating fibres, it cannot be an entire muscle; if, on the other hand, it is an entire muscle, then the fibres must be connected, in which case his objection, that in my operation the effect of the incision by "the fine, sharp, thin knife must necessarily be limited to a simple, momentary separation from each other of two or more adjoining parallel fibres, without any division of their structure," is of no value. But, perhaps, Mr. Nunneley will inform us how (even if the fibres are unconnected) it is possible so to introduce a fine, sharp, thin knife in the course of these fibres, as to cause a simple, momentary separation of *more than two* adjoining fibres, without at the same time dividing structure; and, also, how it is possible to make an oblique incision, of an eighth of an inch long, across the ciliary muscle, (as in my operation), without at the same time cutting through the muscular fibres, even were their arrangement such as described by Mr. Nunneley.

Mr. Nunneley's assertion, moreover, that the fibres of the ciliary muscle are not attached to the schlerotic, is entirely disproved by the investigations of H. Muller, who has traced them from the inner wall of Schlem's canal to their insertion into both schlerotic and choroid. He forgets that a minute portion of the ciliary muscle, teased out upon the object-glass of a microscope, does not represent that muscle as it exists in the natural state; and he forgets, also, the following description of the ciliary muscle, given at p. 175 of his own work on the "Organs of Vision," published in the year 1858:—"If the schlerotic be divided about the third of an inch from the cornea, and the portion with the cornea be carefully detached, we find a whitish-gray circle about four-thirtieths of an inch broad, extending from the junction of the schlerotic and cornea backwards upon the choroid. *It is always a circular belt*, even in those animals, &c. *This substance* has received various names, as ciliary circle or ring, ciliary ligament, ciliary ganglion, and *ciliary muscle*, according to the notions which have been entertained of its structure and functions." It is this whitish-gray circle, this circular belt, this ciliary circle or ring, this ciliary ligament, ganglion, or muscle, which is cut through in the operation which I have proposed. I am ready to admit that if the part did consist merely of a collection of unconnected fibres, the introduction of a knife between, and in the course of, any two fibres would be useless; but where a circle of radiating fibres—I care not how large that circle may be—is rendered a connected whole by circular fibres and other uniting tissue, the solution of that whole by the division of these circular fibres and connecting tissue, even though the radiating muscular fibres are left untouched, must effect a very important influence over the entire circle, especially where from preceding disease that circle has been deprived of its elasticity, and is consequently constricting the parts passing through it, as in the case of the vessels, nerves, &c., passing through the ciliary muscle.

Whilst, as we have seen, some gentlemen have endeavored to depreciate the value of this operation, others have assumed to themselves the credit of originality by altering the direction of the incision. I would submit that these proceedings savour in no slight degree of plagiarism, since in all the principle is the same—the division of the ciliary muscle; and whether this object is attained by making the cut obliquely, as I have recommended, or straight or along the curve of the cornea, it can make but little difference, so long as the muscle is divided and the transparent cornea is not invaded. In the latter case the incision is mostly followed by effusion of blood into the anterior chamber and protrusion of the iris through the wound; and although the blood is usually absorbed in two or three days, the protrusion of the iris causes great and unnecessary pain. These accidents, however, should always be regarded as the fault of the *operator* and not of the *operation*.

This operation has been performed in a wide range of diseases, such as cases of conical cornea, staphyloma corneæ, staphyloma scleroticæ keratitis, posterior staphyloma, amaurosis, irido-choroiditis, and glaucoma. The relief which has resulted from its employment, considered in connection with the anatomical relations of the ciliary muscle, appear to me to prove most incontestably the important influence this muscle must exert in a pathological point of view in certain diseases of the eye, by interrupting the circulation, nervous influence, nutrition and consequent power of reparation of the organ. It is difficult otherwise to account for the results afforded in cases of conical cornea. In one case the projecting cone, attenuated to the greatest degree at its apex, gradually flattened down and resumed the original shape and uniform consistency of the natural cornea. It is equally difficult otherwise to account for its effect in cases of staphyloma of the cornea, a progressive disease, having a great tendency to increase in thickness and consequent projection. In one particular case the operation was followed by gradual absorption of the interstitial deposit and flattening of the cornea, so that the patient, previously unable to close his lids, was relieved from that inability and its consequent deformity and suffering. So likewise in a case of staphyloma sclerotica. Here the pigmental coat of the choroid could be seen through the sclerotica, threatening absorption of that coat, and causing it to bulge so much behind the cornea as to prevent closure of the eyelids. After the operation the bulging subsided, the patient could close the lids without difficulty, whilst the sight of the eye was improved.

Again, in leucoma, or long continued dense opacity of the cornea, we have three cases. In one, the patient, previously blind for nine months, had by the division of the muscle the transparency of the greater portion of the cornea restored, and recovered sufficient sight to enable him to earn his living by cleaning horses, cutting chaff, and following the employment of a coal porter. In another case, the patient, previously blind for six years, recovers the transparency of the cornea, and the power of distinguishing objects; whilst the patient in the third case, who was previously blind for twelve months, and was dismissed from one of our largest hospitals as incurable, had the transparency of the cornea restored so that she was able to read the large type heading a newspaper.

As might be expected, the improvement in these cases is very gradual. In successful cases, the circumference or margin of the leucoma becomes jagged, irregular—as it were, broken up, and at last transparent; and this process continues towards the centre until the cornea becomes clear. When the case has become complicated by the effusion of lymph on the iris and pupil, the transparency of the cornea has in most instances long preceded the absorption of the latter.

Then, again, in blindness from corneal opacity of more recent duration, as in keratitis. Among other cases, was a patient blind for six weeks with complete opacity of the cornea, intense pain and intolerance, at once freed from suffering by the operation, and restored to sight by the gradual recovery of the transparency of the cornea, so that she was able to read the smallest type, do any kind of needle work, and support herself as a nursery governess.

There is not a more striking result of the operation than the almost immediate relief from pain which in many instances succeeds its performance, even in cases where restoration of sight is hopeless; and I consequently often operate for this object solely. For instance, an old lady lost the sight of both eyes from glaucoma in one eye for four years, in the other for eighteen months. She could not see light from darkness, but the pain in her eyes and head was most severe. I divided the ciliary muscle in both eyes, and in three days the pain was gone; and although as I expected, no improvement of sight resulted, she was made happy by the relief afforded. Another patient, in relation to this point, said that something gave way at the time of the operation, when he immediately felt easy.

The cases of posterior staphyloma, with near sight, prove the value of this operation as a means of restoring the "power of accommodation" to the eye. I do not, however, recommend it as a substitute for spectacles, but only in those cases marked by a gradual and persistent shortening of sight, in which glasses afford no assistance; and I would here refer to a case wherein both eyes were operated upon. One was not benefitted, but the other was to so great an extent that, by the patient's own desire, I repeated the operation on the uninjured eye with the happiest results.

In cases of amaurosis the operation has been performed successfully where the eyes have presented the opposite opthalmoscopic appearances of congestion and anæmia of the choroid and retina; and I have been asked upon more than one occasion how I can reconcile this apparent anomaly with what I have advanced as to the influence of the ciliary muscle in disease. "If division of the ciliary muscle relieves and cures a congestion of vessels, intra-ocular pressure, &c., how can it cure atrophy of the same vessels?" It seems to me that the question is at once answered by what I have all along insisted upon—"that the ciliary muscle is not the cause of the original disease, but that it sooner or later becomes affected by that disease;" and that from whatever cause the loss of its elasticity and contractility may arise, the effect upon nutrition and the powers of separation is the same, and its division equally necessary. We can readily, therefore, understand, that in some conditions of constitution, in certain forms of inflammation, or from an accident neglected, a state of low inflammation or passive congestion of the tunics may precede and cause this abnormal state of the ciliary muscle, requiring its division equally as when this state results from that peculiarity of constitution leading to atrophy or fatty degeneration. Nor is it unreasonable to conjecture, that the extent to which the nerves are involved would in no slight degree influence the condition of the blood-vessels.

In irido-choroiditis, this operation is only applicable to recent cases, in which the pupil is free, and there are no adhesions between the iris and capsules of the lens. When the pupil is more or less obliterated, and such adhesions are present, this operation does not hold out the same prospect of relief as iridictomy. But before any operation is performed we should be careful to ascertain, if possible, whether the patient is laboring under any other complaint, particularly one entailing more or less drain upon the system. In one of my cases, the young lady conceded the fact of her having for a long period suffered from hæmorrhoids. When, however, she revealed the fact, and had undergone the necessary operation, the improvement was too great and decided to admit of a doubt of the extent to which the irido-choroiditis was aggravated by the continued loss of blood.

In directing attention to cases of glaucoma, I am now in a position to state that not only has the operation benefitted the patients; but that the benefit has proved persistent—a sufficient answer, I should imagine, to those who, without giving it a trial, have opposed the operation, and asserted that its effects could only be temporary. And, in conclusion, I would briefly refer to the abstract of Mr. Bowman's paper, lately read before the British Association. In a paper which I published in No. 12 of the "Ophthalmic Hospital Reports," I expressed the belief that the success of irridectomy depended upon the division of the ciliary muscle, and that in process of time it would be found that the extent of the incision hitherto practised in that operation might be materially diminished, and the tearing away of the iris altogether dispensed with. Although Mr. Bowman has not paid me the compliment of noticing this opinion, I am happy to find from his paper that his views are approximating to it, and that, abandoning his original conjectures as to the *modus operandi* of iridectomy, he now states, "As to the action of irridectomy in relieving glaucoma, he did not wish to speak confidently. It might be the removal of part of the secreting surface of the iris, but it appeared that it was *not due so much to the quantity removed as to the exactitude with which it was removed at its attached border.*"

---

*Alkaloids and Alcohol.*—A Cabinet order, published in the Prussian *Moniteur*, directs that the brandy served out in the Prussian army shall be henceforth replaced by coffee. Each man will receive two-fifths of an ounce per day in time of peace, and half an ounce in time of war.

*Treatment of Chronic Dysentery with Ipecacuanha.* By Dr. WILLSHIRE, of Charing-Cross Hospital.

The first essential thing was stated to be the plabiny of the patient in the most complete state of repose possible, and not allowing for one moment the intestines to drag down by their own weight, and thus to excite, or at any rate to increase, the tenesmus and tormina. Hence the patient must be rigorously confined to bed, and not allowed to rise even for the purpose of evacuating the bowels. The latter object must be effected by means of the bed-pan. Under no entreaties is the patient to be permitted to rise at first, even when he asserts he is much better. The second object is to give the bowels as little to do as possible in the way of being called upon to evacuate the unconvertible and refuse material of diet. Hence such food alone must be given as leaves the least amount of excrementitious matter behind it. A baby's diet is the one to be adopted. Milk, eggs, isinglass, gelatine, rice, and analagous non-irritating articles, must be adhered to most strictly. Such things, whilst they are nutritious, make but little fœcal matter which can irritate, in its passage through the intestinal canal, the already irritable bowels. The next thing is to apply gentle counter-irritation and warmth to the abdominal surface. They may be alternated one with the other, or used singly, or repeated for a time according to circumstances. Lastly, as far as his (Dr. Willshire's) experience went, the remedial treatment should consist mainly of the combination of opium with certain gastro-intestinal sedatives and astringents, viz: bismuth, bael, ipecacuanha, and logwoods. Of bael and ipecacuanha a very high opinion had been formed by many and experienced Indian practitioners. He himself had used the former in several hospital and in one or two private cases, and had formed a good opinion of its powers in checking what he should prefer to consider as chronic diarrhœa or dysenteric catharsis rather than as analogous to true chronic dysentery. In the case cited, it would be seen that the bael, though apparently affording relief at first, failed to continue it, and that a relapse was the consequence. It was not until what some have deemed almost a specific for dysentery—viz: ipecacuhana was administered, that marked and permanent improvement followed, the blood disappearing from the stools, the mucous becoming less in amount, and finally the dejections appearing consistent. The ipecacuanha had here been employed in small doses only. Had these not been followed by good effects, he should have given the usual larger ones, such as twenty-five grains or half a drachm every four or six hours. But as ten grains three times in the day seemed to be sufficient, he had left well alone, and it was seen with good consequences. The extract of hæmatoxylon had been employed in this case, and, speaking generally, he thought it well adapted for disenteric catharsis. He had got into the habit of employing it from witnessing its good effects in the milder forms of "entero-colitis" in children. In those little patients, the diarrhœa often became dysenteric, and indeed bad cases of it were really those of true dysentery—in other words, entero-colitis. Suffice it to say, in such like instances, hæmatoxylon acted as a sedative astringent, or as one perfectly devoid of any irritant character, and would be frequently found to be followed by a diminution of intestinal discharge, when other drugs either produced no such effect, or seemed to make it more copious or frequent. One or two cases had been recorded in which the arrest of chronic diarrhœa by hæmatoxylon had been followed by *phlebitis* of the veins of the lower extremities, and such a result was regarded as specifically belonging to the action of logwood. He himself was quite ignorant of any ill effect from its use.

---

*On the Arcus Senilis.*—This is the occurrence of a little band of pearly whiteness around the edge of the cornea, which had long been noted and named, but was little thought of either by surgeons or patients. Mr. Canton has ascertained that this change, so frequent in old age, is due to "fatty degeneration" of the corneal tissue. Immediately that the pathological characters of this change were discovered, it was possible to determine the relation in which it was likely to stand to similar changes in other and concealed organs. For this is the privilege which observers possess in watching the progress of disease in the eye, or in interpreting the significance of its pathological alterations, that from the seen they may argue to the unseen; and so the pathology of the eye may afford the best light for aiding in the diagnosis of disease in other parts of the system. Mr. Canton set himself to determine the semilogical value of this fatty degeneration; and as to the success of his search we may quote the verdict of M. Paget, who says: "The arcus seems to be, on the whole, the best indication which has yet been found of proneness to an extensive or general fatty degeneration of the tissues. It is not, indeed, an infallible sign thereof; for there are cases in which it exists with clear evidence of vigor in the nutrition of the rest of the body; and there are others in which its early occurrence is due to defective nutrition consequent on purely local causes, such as inflammatory affections of the choroid, or other parts of the eye. But, allowing for such exceptions, it appears to be the surest, as well as the most visible sign, and measure of these primary degenerations." Mr. Canton adopts this view, and does not desire to place a more positive or dogmatic interpretation on the sign which he has pointed out, than facts give it. Dr. Watson, in his "Practice of Physic," in speaking of the arcus, observes: "The cornea is sometimes alone in suffering the change. I am acquainted with a gentleman, under forty years of age, who, enjoying excellent health, presents a well-pronounced arcus in both eyes, especially at the summit and at the base of the circle, and in whom that appearance has remained unaltered, certainly since he was twenty-four years old, and perhaps from an earlier date. But Mr. Canton calls to mind that the apparent enjoyment of excellent health is no argument in favor of the non-existence of fatty degeneration of the heart; and in proof of this state refers to recent notable deaths, in which the autopsy revealed the existence of fatty degeneration of the heart, although excellent health had previously been enjoyed. No doubt it is important that the frequent exceptions to this coincidence of general fatty degeneration with the "arcus" should be borne well in mind, for otherwise a considerable source of hypochondriacal fears would be added to those which already torment the world. But the arc occurring in young persons or adult men is a sign—a rainbow in the sky—which the surgeon or physician should not neglect.

*Topical Injections of Strychnine.*—M. Courty, Professor of Surgery at the Faculty of Montpelier, having succeeded in controlling severe neuralgic pains by injections of strychnine, tried them likewise in paralysis of the facial nerve, as well as the loss of power of movement in other parts. In different cases of paralysis, and especially in chronic cases, the result was most favorable. The author, succeeded, however, in a case of paraplegia; the patient, a woman aged forty-five, having been thus paralyzed for twelve months, many remedies had been tried; but a few injections of strychnine on a level with the inferior extremity of the spinal marrow sufficed for the cure. Success was also obtained in three cases of recent facial paralysis. The first patient was a man of fifty-six; the second, a lady of twenty-five, and the third a young lady of twenty-two. They were all in the early stage of the disease; and the strength of the solution varied from one in a hundred to one in seventy. A few drops (from eight to sixteen) were injected along the course of the facial nerve, between the stylo-mastoid foramen and the neck of the lower maxilla. The injection was repeated every second or third day. All the muscles of the face recovered the faculty of movement after from three to six injections, in about ten days or a fortnight. The author states that no relapses have taken place in these cases.

*Chylous Urine Successfully Treated by Tincture of Muriate of Iron.*—Dr. Dutt relates the case of a Hindoo, aged twenty-four years, who reported himself to have been suffering from chylous urine for four years, during which time he had been under several practitioners, and had taken a variety of medicines, but with very little benefit. On examining a specimen of his urine it was found to be of a milky-white color, thick, and full of coagula. There was no pinkish tinge in it. It was not examined for sugar. The patient stated that at the commencement he had slight pain in the region of the right kidney, but that it was removed by a blister, which, however, did not produce any effect on his morbid urine. He was dyspeptic; but his general health was good, and there was no evidence of any visceral disease or any local affection of the lymphatic system. The peculiarities of this case were that the urine passed during the day was clear and free from chyle, while that voided during the night and in the morning was deeply loaded with it. At night micturition was frequent, and the urethra would sometimes get blocked up by coagula. He was very much subject to night emissions. He was at first treated for dyspepsia, which did nothing more than improve his appetite. For this purpose he was given tonics, anti-acids, &c. Gallic acid was then tried, in three grain doses, three times a day, for five days, but with no better success. In fact, the symptoms were aggravated while under this treatment. He was then ordered fifteen minims of tincture of muriate of iron, in an ounce of infusion of quassia, to be taken three times a day. Before he had taken the medicine for three days the improvement in his urine was marked, and at the end of the week it was entirely free from chyle. He was kept under observation for more than four weeks after this, and the drug intermitted for a week, and his urine continued free from chyle. He was discharged cured. There are one or two points in connection with this case worthy of note: Was there any connection as cause and effect between the dyspepsia and the chylous urine? This question must be answered by others; but it is quite evident that there could not have been any organic lesion excepting a slight irritation of the right kidney at the outset. The night emissions may have been due to some irritation existing at the mouth of the bladder; the patient remained subject to them long after the urine had ceased to present any trace of chyle in it.

---

*What is Carbolic Acid?*—Dr. Frodsham, writing to the *Lancet*, says: "In reading the remarks made by Dr. Calvert and other gentlemen, describing the therapeutic properties of carbolic acid, one would be led to believe in this as a new therapeutic agent, and that this substance possessed some peculiar medicinal properties inherent in itself, whereas, under the name of creasote it has been used extensively in medicine for years; pure German creasote being identical, medically and chemically, with carbolic acid, the chief difference seeming to be that carbolic acid can be obtained in crystals, which, however, on contact with the air, assume the liquid form." Professor Gregory says: "So great is this resemblance, that I am inclined to consider creasote as a somewhat impure carbolic acid, the impurities being substances homologous with carbolic acid, or rather the carbolic acid is the impurity in a body belonging to the same homologous series; the taste, smell, density, boiling point, solubility in water, poisonous and antiseptic action of these two bodies as the same. These results I have myself also obtained, and it would appear that if creasote be not carbolic acid contaminated with some foreign matter, these two bodies are, at least, closely connected and belong to the same series, which is either that of benzole or that of phenyle? (*vide* Gregory's 'Handbook of Organic Chemistry.) Perhaps Dr. Calvert will kindly inform us whether his researches have thrown any additional light on the investigations of Professor Gregory and Liebig as to the chemical composition of this substance, and also in what manner its medicinal properties differ from those of creasote?"

---

*Extreme Mobility of both Kidneys.*—Mary J——, aged forty-two, married, the mother of ten children. For the last ten years the patient has felt a tumor in the right side of the abdomen, about the size of a turkey's egg; and within the past few years she became aware that a similar tumor existed in the left side. Sometimes she was not able to discover either tumor, and therefore imagined that the swelling moved from side to side, or that it disappeared at times. She has never suffered pain or other symptom in connection with the tumors, nor has any increase in size taken place. During the last few years she has become extremely nervous and excitable, passing sleepless nights, imagining she was the subject of some serious disease. Upon examination, a tumor is easily discovered in each lumbar region upon relaxation of the abdominal parieties. Their form and size resemble the kidneys, smooth on the surface, with a distinct rounded and hollowed border. When the hand is placed upon either tumor, no pain is felt, but upon rather firm pressure, a peculiar sickly sensation is complained of. These tumors are distinctly movable, especially downwards and forwards, in the latter direction as far as the outer border of the rectus abdominis muscle. During the examination, the tumors once or twice glided away, and were again to be sought for. The urine perfectly normal, the lungs and heart healthy. The patient is very nervous and apprehensive of her condition. She was assured that no disease existed; and that these supposed tumors were to her a natural condition, being an "extreme mobility of the kidneys." A change of air and scene was recommended, with the administration of a mild tonic.—*Lancet.*

